# Predictive value of baseline thyroid autoantibody titers for ICI-associated thyroid adverse events in cancer

**DOI:** 10.1530/ETJ-26-0017

**Published:** 2026-04-29

**Authors:** Li Jian, Qin Ou, Taiyu Xia, Guhang Tang, Ya Yu, Qiquan Zhao, Qinan Wu

**Affiliations:** ^1^ Department of Endocrinology, The Affiliated Dazu’s Hospital of Chongqing Medical University, The People’s Hospital of Dazu, Chongqing, China; ^2^ Department of Oncology, The Affiliated Dazu’s Hospital of Chongqing Medical University, The People’s Hospital of Dazu, Chongqing, China; ^3^ Department of Respiratory and Critical Care Medicine, The Affiliated Dazu’s Hospital of Chongqing Medical University, The People’s Hospital of Dazu, Chongqing, China

**Keywords:** immune checkpoint inhibitors, thyroid immune-related adverse events, thyroid autoantibodies

## Abstract

**Objective:**

To investigate the predictive value of pretreatment thyroid autoantibody titers for thyroid immune-related adverse events (irAEs) in patients receiving immune checkpoint inhibitors (ICIs).

**Methods:**

In this study, 181 patients with malignancies treated with ICIs between January 2022 and October 2025 were retrospectively analyzed. The patients were categorized based on the occurrence of thyroid irAEs. Risk factors and the impact of antibody titers were analyzed using logistic regression and Kaplan–Meier methods.

**Results:**

Thyroid irAEs occurred in 44 (24.3%) patients, predominantly including hypothyroidism (81.8%). Logistic regression revealed that baseline thyroid-stimulating hormone (TSH) levels, thyroid peroxidase antibody (TPOAb) positivity, and thyroglobulin antibody (TgAb) positivity were significant risk factors (*P* < 0.05). Kaplan–Meier analysis revealed a significantly higher incidence of irAEs in patients with elevated baseline TPOAb and TgAb titers than in those with normal titers (*P* < 0.01). Notably, severely elevated TPOAb titers resulted in a significantly higher risk of irAEs than moderately elevated titers (*P* < 0.05), whereas the corresponding difference for TgAb titers was not significant.

**Conclusion:**

Baseline TPOAb and TgAb titers are effective predictors of thyroid irAEs. Higher TPOAb titers are correlated with an increased risk, thereby supporting the utility of quantitative baseline screening for risk stratification.

## Introduction

ICIs have achieved breakthroughs in tumor treatment because of their unique mechanisms of action. They primarily function by blocking various signaling pathways, such as those involving programmed cell death protein 1 (PD-1)/programmed cell death ligand 1 (PD-L1), cytotoxic T-lymphocyte-associated protein 4 (CTLA-4), and lymphocyte-activation gene 3 (LAG3). By inhibiting the interaction between tumor cells and the immune system, ICIs enhance the immune system’s ability to attack tumor cells (1). However, this mechanism may also induce the immune system to attack normal host tissues and organs, thus, leading to irAEs that can affect multiple organ systems throughout the body, primarily the skin and mucous membranes, musculoskeletal system, endocrine system, immunologic system, digestive system, cardiovascular system, respiratory system, and nervous system (2, 3). Among these events, thyroid irAEs have emerged as a major clinical concern because of their relatively high incidence, insidious onset, potential to cause irreversible thyroid dysfunction, risk of delaying or interrupting antitumor treatment, and, in rare instances, life-threatening consequences (4).

Previous studies have reported that women with TPOAb and normal TSH levels have a 2.1% annual risk of developing clinical hypothyroidism, and this risk is correlated with antibody titers; specifically, the risk is 4% in antibody-negative individuals, 23% in those with weakly positive antibodies, 33% in those with moderately positive antibodies, and 53% in individuals with strongly positive antibodies (5). Currently, the research on risk predictors for ICI-associated thyroid irAEs in patients with malignancies indicates that patients exhibiting positivity for thyroid autoantibodies (specifically TPOAb and/or TgAb) have an increased risk of developing thyroid irAEs (4, 6, 7). However, existing studies have predominantly focused on the association between the ‘positive/negative’ status of antibodies and the risk of thyroid irAEs. It remains unclear whether the risk of thyroid irAEs is correlated with the specific baseline titer levels of TPOAb and TgAb. Furthermore, evidence and strategies for the stratified management of thyroid irAEs in patients with malignancies based on thyroid autoantibody titer levels are lacking. Therefore, this study aimed to investigate the association between baseline TPOAb and TgAb titers and the risk of thyroid irAEs. The patients were categorized into normal, moderately elevated, and severely elevated groups, and the risk of thyroid irAEs was compared among the three groups.

## Subjects and methods

### Study subjects

The study subjects consisted of cancer patients who were hospitalized at our institution between January 2022 and October 2025. The inclusion criteria were as follows: i) received treatment with ICIs; ii) completed relevant thyroid function assessments; and iii) aged between 18 and 80 years.

**Figure 1:**
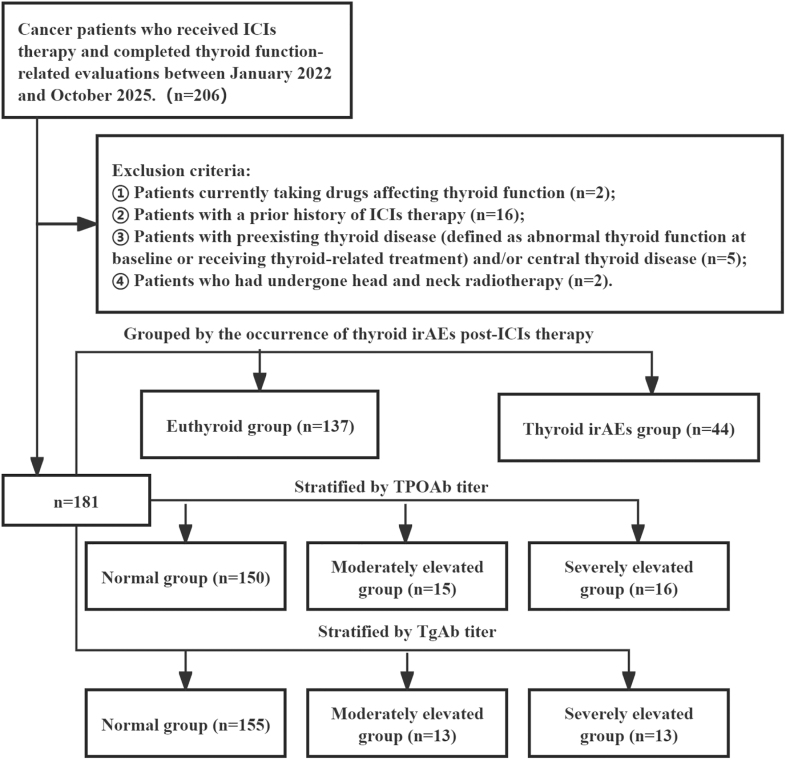
Flow chart of study subject inclusion, exclusion, and group distribution.

The exclusion criteria were as follows: i) concurrent use of medications affecting thyroid function (e.g. amiodarone, glucocorticoids, or lithium preparations); ii) prior exposure to ICI treatment; iii) preexisting thyroid disease (defined as abnormal baseline thyroid function or the receipt of thyroid-related medication) and/or central thyroid disease; and iv) a history of head and neck radiotherapy (Fig. 1). This study was approved by the Ethics Committee of the Affiliated Dazu’s Hospital of Chongqing Medical University (Approval No. 2024LLSC024). The requirement for informed consent was waived because of the retrospective nature of the study.

### Methods

Patients receiving ICI therapy were routinely hospitalized for safety monitoring during the first few treatment cycles. Clinical data, tumor types, ICI drug types, thyroid function, and thyroid autoantibody (TPOAb and TgAb) titers of cancer patients before and after ICI treatment were collected. The time of thyroid irAE onset was also recorded. TPOAb and TgAb titers were detected via the Atellica^®^ IM chemiluminescence immunoassay system (Siemens Healthineers, Germany) and the corresponding reagent kits. The thresholds for antibody positivity for TPOAb and TgAb were defined as >60 IU/mL and >4.5 IU/mL, respectively. All patients received at least one cycle of ICI treatment. Serum levels of free triiodothyronine (FT3), free thyroxine (FT4), and TSH were assessed every 3–4 weeks after treatment, with at least two thyroid function follow-up assessments. The median follow-up duration was 8 months, with a range of 6–49 months.

### Outcome measures and evaluation criteria

The patients were stratified into a euthyroid group and a thyroid irAE group based on the occurrence of thyroid irAEs following ICI treatment. The relationships between the risk of thyroid irAEs and variables such as age, sex, ICI type, tumor type, baseline TSH level, and baseline thyroid autoantibody titers were evaluated. Given that no uniform quantitative thresholds are available for determining the risk of thyroid irAEs in existing guidelines and that detection methods and reference ranges vary across institutions, the median-split method was used for exploratory analysis. The patients were divided into a normal group (titers below the upper limit of normal) and moderately and severely elevated groups (based on median values of antibody titers). The median titers for TPOAb and TgAb were 175.4 IU/mL and 38.7 IU/mL, respectively, which served as cutoff values for grouping. The patients were divided into three groups based on their TPOAb titers: the normal group (TPOAb titer: <60 IU/mL, i.e. below the upper limit of normal (ULN)), the moderately elevated group (TPOAb titer: 60 – 175.4 IU/mL), and the severely elevated group (TPOAb titer: ≥ 175.4 IU/mL). Similarly, the patients were grouped based on their TgAb titers as follows: the normal group (TgAb titer: < 4.5 IU/mL, i.e. below the ULN), the moderately elevated group (TgAb titer: 4.5 – 38.7 IU/mL), and the severely elevated group (TgAb titer: ≥ 38.7 IU/mL). Differences in the incidence of thyroid irAEs among these groups were observed. Thyroid irAEs included thyrotoxicosis (elevated FT3 and FT4 levels; a decreased TSH level), subclinical thyrotoxicosis (normal FT3 and FT4 levels; TSH level < lower limit of normal), hypothyroidism (decreased FT3 and FT4 levels; an elevated TSH level), and subclinical hypothyroidism (normal FT3 and FT4 levels; ULN < TSH < 10 mIU/L).

### Statistical analysis

Data analysis was performed via the GraphPad Prism 10.1.2 software. Categorical data are expressed as *n* (%) and were analyzed with the chi-square (*χ*^2^) test. Continuous data are expressed as the mean ± standard deviation (*x* ± s) and were analyzed using a *t*-test. Nonnormally distributed continuous data are expressed as medians (Q1, Q3), and group comparisons were performed using the Mann–Whitney *U* test. A *P* value <0.05 was considered to indicate statistical significance. Multivariate logistic regression was used to analyze the risk factors for thyroid irAEs. Kaplan–Meier curves were constructed to analyze the impact of thyroid autoantibody titers on the occurrence of thyroid irAEs.

## Results

### Comparison of clinical characteristics of patients with malignancies treated with ICIs between the euthyroid group and the thyroid irAE group

For the 181 patients with malignancies treated with ICIs, the median number of treatment cycles was 8. Thyroid irAEs occurred in 44 patients (24.30%). Among these, 36 patients (81.81%) presented with clinical or subclinical hypothyroidism, while 8 patients (18.18%) presented with clinical or subclinical thyrotoxicosis. Subsequently, 5 patients with thyrotoxicosis progressed to hypothyroidism. In total, 41 patients (93.18%) eventually developed hypothyroidism. Baseline TSH levels of patients in the thyroid irAE group were significantly higher compared with those of patients in the euthyroid group (*P* < 0.01). With respect to tumor types, patients with liver cancer were more likely to develop thyroid dysfunction (*P* < 0.05). For patients in the euthyroid group, the positivity rates of both TPOAb and TgAb were 5.11%, whereas for patients in the thyroid irAE group, the positivity rates were 54.55 and 43.18%, respectively. The difference in autoantibody positivity rates between the two groups was statistically significant (*P* < 0.01). No significant differences were observed between the two groups regarding clinical variables, including sex, age, type of ICIs, and the number of treatment cycles (*P* > 0.05) (Table 1).

**Table 1:** Comparison of clinical characteristics of 181 patients with malignancies treated with ICIs between the euthyroid and thyroid irAE groups. Data are presented as *n* (%) or as median (Q1, Q3).

Parameters	All	Euthyroid group	Thyroid irAE group	*χ*^2^/Z	*P*
*n*	181	137	44		
Sex				2.657	0.103
Male	152 (83.98)	119 (86.86)	33 (75.0)		
Female	29 (16.02)	18 (13.14)	11 (24.0)		
Age (years)	66.00 (58.00, 72.00)	66.00 (59.00, 72.00)	64.00 (57.50, 69.00)	0.949	0.343
Number of ICI treatment cycles	8 (4, 16)	6 (3, 10)	7.5 (3, 15.5)	0.898	0.370
Baseline TSH (mIU/L)	1.82 (1.14, 2.76)	1.61 (1.02, 2.28)	2.73 (1.87, 3.61)	−4.632	<0.001
Tumor type				8.301	0.081
Lung cancer	88 (57.89)	76 (55.47)	12 (27.27)	2.28	0.118
Liver cancer	46 (25.41)	30 (21.9)	16 (36.36)	3.34	0.048
Esophageal cancer	13 (7.18)	10 (5.52)	3 (6.82)	0.14	0.767
Nasopharyngeal carcinoma	11 (6.07)	6 (4.38)	5 (11.36)	1.75	0.139
Others	23 (12.7)	15 (10.95)	8 (18.18)	0.34	0.523
TPOAb positivity	31 (17.13)	7 (5.11)	24 (54.55)	53.91	<0.01
TgAb positivity	26 (14.36)	7 (5.11)	19 (43.18)	36.21	<0.01
Type of ICI				4.865	0.301
Tislelizumab	76 (41.99)	53 (38.69)	23 (52.27)	2.524	0.112
Camrelizumab	30 (16.57)	25 (18.25)	5 (11.36)	1.142	0.285
Toripalimab	29 (16.02)	25 (18.25)	4 (9.09)	2.076	0.150
Sintilimab	17 (9.39)	12 (8.76)	5 (11.36)	0.265	0.606
Other ICIs or CT	29 (16.02)	22 (12.15)	7 (15.91)	0.001	0.981
Clinical or subclinical					
Thyrotoxicosis	8 (4.42)	-	8 (18.18)		
Hypothyroidism	36 (19.89)	-	36 (81.82)		

irAE, immune-related adverse event; TSH, thyroid-stimulating hormone; TPOAb, thyroid peroxidase antibody; TgAb, thyroglobulin antibody; ICI, immune checkpoint inhibitor; ‘-’, no specific value; CT, combination therapy.

### Logistic regression analysis of risk factors for the occurrence of thyroid irAEs following ICI treatment

A logistic regression analysis was performed with the occurrence of thyroid irAEs following ICI treatment in patients with malignancies as the dependent variable and potential risk factors as independent variables. The results indicated that baseline TSH levels, baseline thyroid autoantibody positivity, baseline TPOAb positivity, and baseline TgAb positivity were risk factors for the occurrence of thyroid irAEs (*P* < 0.01). No statistically significant associations were observed for age or sex (*P* > 0.05) (Table 2).

**Table 2:** Logistic regression analysis of risk factors for the occurrence of thyroid irAEs following ICI treatment.

Independent variables	*Β*	SE	Wald *χ*^2^	OR (95%CI)	*P* value
Age (<66 years)	0.285	0.348	0.67	1.32 (0.67–2.63)	0.413
Sex (male)	−0.790	0.430	3.37	0.45 (0.20–1.05)	0.066
TSH (>1.82 mIU/L)	1.438	0.389	13.66	4.21 (1.96–9.02)	<0.001
ThAAb positivity	2.711	0.431	39.66	15.05 (6.47–34.99)	<0.001
TPOAb positivity	3.104	0.492	39.78	22.29 (8.49–58.47)	<0.001
TgAb positivity	2.647	0.493	28.82	14.11 (5.37–37.10)	<0.001

ThAAb, thyroid autoantibody.

Note: The dependent variable was the occurrence of thyroid irAEs (occurrence = 1; nonoccurrence = 0). Variable definitions: age: <66 years = 1, ≥66 years = 0; sex: male = 1, female = 0; TSH level: >1.82 mIU/L = 1, ≤1.82 mIU/L = 0; thyroid autoantibody variables: positivity for any thyroid autoantibody, TPOAb, or TgAb = 1, negative = 0.

### Comparison of the cumulative incidence of thyroid irAEs stratified by TPOAb and TgAb titers

**Figure 2:**
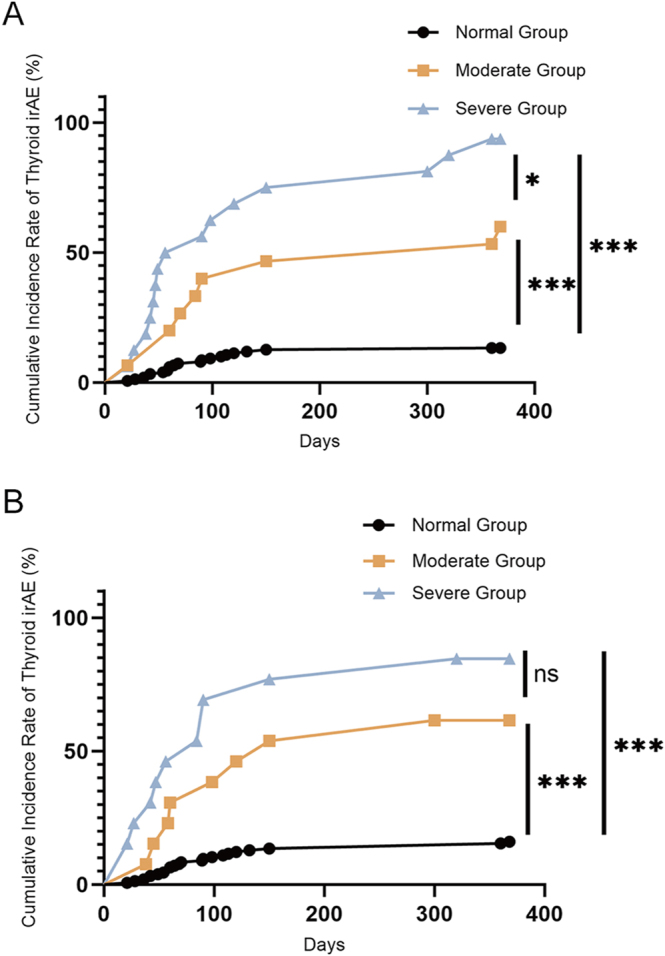
(A) Cumulative incidence of thyroid irAEs stratified by different TPOAb titer groups (Kaplan–Meier curves). Note: panel A displays the Kaplan–Meier curves for the cumulative incidence of thyroid irAEs among patients across the three groups having different titers of TPOAb. *P = 0.037; ***P < 0.001. (B) Cumulative incidence of thyroid irAEs stratified by different TgAb titer groups (Kaplan–Meier curves). Note: Panel B displays the Kaplan–Meier curves for the cumulative incidence of thyroid irAEs among patients across the three groups having different titers of TgAb. ***P < 0.001.

The incidence of thyroid irAEs was 13.33% (20/150) among patients in the baseline normal TPOAb group, 60.0% (9/15) among patients in the moderately elevated TPOAb titer group, and 93.75% (15/16) among patients in the severely elevated TPOAb titer group (Table 3). Compared with incidences among patients in the normal group, the differences in incidences among patients in both the moderately and severely elevated TPOAb titer groups were statistically significant (*P* < 0.001). The incidence of thyroid irAEs among patients in the severely elevated group was significantly greater than that among patients in the moderately elevated group (*P* < 0.05) (Fig. 2A). The incidence of thyroid irAEs was 16.13% (25/155) among patients in the baseline normal TgAb group, 61.54% (8/13) among patients in the moderately elevated TgAb titer group, and 84.62% (11/13) among patients in the severely elevated TgAb titer group (Table 3). Comparisons of incidences among patients in the moderately and severely elevated TgAb titer groups with those among patients in the normal group revealed statistically significant differences (*P* < 0.001). Although the incidence of thyroid irAEs among patients in the severely elevated group was higher than that among patients in the moderately elevated group, the difference between the two groups was not statistically significant (Fig. 2B).

**Table 3:** Incidence of thyroid irAEs stratified by different antibody titer groups.

Groups	Total (*n*)	irAEs (*n*)	Incidence
TPOAb titers			
Normal group	150	20	13.33%
Moderately elevated group	15	9	60.0%
Severely elevated group	16	15	93.75%
TgAb titers			
Normal group	155	25	16.13%
Moderately elevated group	13	8	61.54%
Severely elevated group	13	11	84.62%

## Discussion

The incidence of thyroid irAEs among patients with malignancies treated with ICIs varies widely, ranging from 6.1 to 44.4% (8, 9). Excluding subclinical thyroid irAEs, the incidence of overt thyroid irAEs ranges from 8.9 to 22.2% (10, 11, 12, 13). Some studies have reported that the occurrence of thyroid irAEs is associated with improved progression-free survival (PFS) and overall survival (OS) in tumor patients (14, 15). The typical clinical course involves biphasic changes in thyroid function; specifically, transient thyrotoxicosis appears 2–6 weeks after treatment initiation, followed by persistent hypothyroidism at approximately 12 weeks. Moreover, approximately 79.2% of patients require long-term levothyroxine replacement therapy (3, 16). Rare instances of Graves’ disease and thyroid-associated ophthalmopathy have also been reported (17). In the present study, the incidence of thyroid irAEs among patients following ICI treatment was 24.30%, and no grade III–V thyroid irAEs were observed. At the time of diagnosis, 81.81% of the patients presented with clinical or subclinical hypothyroidism, while 18.18% of the patients presented with clinical or subclinical thyrotoxicosis. Notably, the majority of patients with thyrotoxicosis subsequently progressed to hypothyroidism. Consequently, 93.18% of all thyroid irAEs ultimately evolved into hypothyroidism. A previous study reported that all patients with baseline TPOAb positivity who developed thyrotoxicosis subsequently progressed to hypothyroidism (18). These findings suggest that baseline TPOAb positivity may serve as a potential marker for the subsequent development of hypothyroidism in patients who present with thyrotoxicosis.

The exact mechanisms underlying the development of irAEs in patients with malignancies treated with ICIs remain unclear. Current hypotheses primarily point toward the following factors: irreversible damage caused by organ-specific inflammation (manifesting as organ injury and/or dysfunction); prolonged inflammatory toxicity causing organ damage (3); cross-reactivity between tumor antigens and self-antigens (19); changes in microbiome composition (20); and lingering autoimmunity (18), as well as genetic and environmental factors. By blocking the PD-1/PD-L1 signaling pathway, ICIs increase T-cell immune activity, thereby increasing the likelihood of T-cell attacks on host tissues (21) Yasuda *et al.* established a mouse model of thyroid irAEs in which histopathological analysis of thyroid tissue revealed CD4+ T-lymphocyte infiltration, thereby suggesting that CD4+ T lymphocytes may play a crucial role in the development of irAEs (22). Torimoto *et al.* reported that PD-1 and PD-L1 inhibitors can increase the proportion of follicular helper T cells. Tfh cells regulate B-lymphocyte maturation, activation, and antibody production, thus participating in the pathogenesis of autoimmune diseases (23). Melissa G *et al.* reported that various type 3 immune cells (such as γδT17, CD4+Th17, and CD8+ Tc17 cells) participate in the development of thyroid irAEs (4). Furthermore, ICIs may induce autoimmune thyroiditis through other mechanisms, such as by altering the immunogenicity of thyroid cells or increasing autoantibody production (24). In patients with thyroid irAEs, fine-needle aspiration (FNA) of the thyroid tissue has revealed lymphocyte accumulation similar to that observed in Hashimoto’s thyroiditis (25). Although thyroid irAEs share similarities with autoimmune thyroid diseases, it remains unclear as to whether they represent the same syndrome (2). Clinically, the onset and progression of thyroid irAEs are significantly faster than those of classical autoimmune thyroid diseases.

Studies have identified several potential risk factors for thyroid irAEs, including preexisting thyroid disease, thyroid autoantibody positivity, baseline TSH levels, high uptake of 18F on thyroid 18F-FDG-PET, a heterogeneous appearance on thyroid ultrasound, prior use of tyrosine kinase inhibitors (TKIs), and body mass index (BMI) (13, 26, 27). Baseline thyroid autoantibody positivity is an independent predictor of thyroid irAEs; in particular, TgAb positivity is a significant risk factor (18, 28). Previous studies have reported that the positivity rates of TPOAb and TgAb were markedly higher in patients who eventually developed thyroid irAEs after ICI treatment (21 and 32%, respectively) compared with those of patients who did not develop irAEs (only 3%) (11). The results of this study revealed that the positivity rates of TPOAb and TgAb were 54.55 and 43.18%, respectively, among patients in the thyroid irAE group, whereas the positivity rates of both antibodies were 5.11% among patients in the euthyroid group. Among these, the positivity rate of TPOAb among patients with thyroid irAEs was higher than that reported in previous studies. The risk of developing thyroid dysfunction is 1–2.5% in patients exhibiting TPOAb and TgAb negativity, whereas it increases to 20–50% in antibody-positive patients (8, 26). A 20-year community-based follow-up study of thyroid disease conducted by Wickham *et al.* revealed that the annual risk of clinical hypothyroidism among TPOAb-positive women was 2.1%, and the risk increased with increasing antibody titers; specifically, the risks were 4% for antibody-negative patients, 23% for weakly positive patients, 33% for moderately positive patients, and as high as 53% for strongly positive patients (5). The present study demonstrated that hypothyroidism progressed rapidly in patients after treatment with ICIs, with a median onset time of 69 days. The incidences of hypothyroidism among patients in the normal, moderately elevated, and severely elevated TPOAb groups were 12.67, 53.33, and 87.5%, respectively, which were significantly higher than those observed among individuals in the general community population. Christopher A *et al.* reported that elevated titers or seroconversion of TgAb and TPOAb during ICI treatment were associated with thyrotoxicosis. Potential explanations for this include i) PD-1 pathway blockade, which induces T-cell-dependent B-cell activation and autoantibody secretion (29), and ii) thyroiditis, which leads to the release of thyroid antigens, subsequently triggering humoral immunity and autoantibody production. These findings suggest that elevated TPOAb and TgAb titers may serve as biomarkers for identifying adverse thyroid immune-related events (30). The results of this study revealed that the cumulative incidence of thyroid irAEs in patients with moderately and severely elevated baseline TPOAb and TgAb titers was consistently higher than that among patients in the normal group. Notably, the incidence of thyroid irAEs was significantly greater among patients in the severely elevated TPOAb group than that among patients in the moderately elevated group. These results indicate a dose–response relationship; specifically, higher TPOAb titers are correlated with a higher incidence of ICI-associated thyroid irAEs. Therefore, a stratified management strategy based on TPOAb titers is proposed for clinical practice. For patients with significantly elevated TPOAb titers, follow-up monitoring of clinical symptoms should be intensified, and the frequency of thyroid function testing should be increased to achieve early identification of and intervention for thyroid dysfunction. Multiple clinical studies have demonstrated that selenium supplementation can reduce thyroid autoantibody levels and decrease the incidence and symptom severity of autoimmune thyroiditis (31, 32). Selenium supplementation may reduce TPOAb titers and the risk of thyroid irAEs in patients with markedly elevated TPOAb levels who are receiving ICI therapy, which warrants investigation in future studies.

This study has several limitations: it was retrospective in design with no available mature survival data; it included a variety of cancer types and ICI regimens; it was conducted at a single center; and it included a relatively small number of patients demonstrating positivity for thyroid autoantibodies. Therefore, further studies with larger patient cohorts are needed to clarify the quantitative relationship between TPOAb and TgAb titers and the occurrence of thyroid irAEs.

## Conclusion

As shown in this work, baseline positivity for TPOAb and TgAb constitutes risk factors for ICI-associated thyroid irAEs. Further stratified analysis revealed a significant positive correlation between TPOAb titers and the incidence of thyroid irAEs, thus demonstrating a clear dose–response relationship. Based on these findings, for the population receiving ICI therapy with significantly elevated TPOAb titers, a precise stratified management strategy is recommended involving the intensification of dynamic follow-up monitoring of clinical symptoms and an increased frequency of thyroid function tests. This study provides important evidence-based support for optimizing individualized ICI treatment regimens and enhancing the clinical safety of tumor immunotherapy.

## Declaration of interest

The authors declare that there are no conflicts of interest that could be perceived as prejudicing the impartiality of the reported research.

## Funding

This research was supported by the Scientific and Technological Research Program of Chongqing Municipal Education Commission (Grant No. KJQN202400406), the Science and Technology Development Program of Chongqing Dazu District (DZKJ2024JSYJ-KWXM1042), the General Medical Research Project of Chongqing Science and Health Joint Medical Research (2026MSXM024), the Chongqing Medical Leading Talent Project (YXLJ202514), and the Chronic Disease Management Research Project of National Health Commission Capacity Building and Continuing Education Center (GWJJMB2010024191).

## Author contribution statement

LJ and QO wrote, reviewed, and edited the manuscript. TYX conducted the research. QQZ and QNW analyzed the data. TYX, GHT, and YY provided technical assistance and contributed to the discussion. LJ, QO, QQZ, and QNW designed and directed the project and contributed to the discussion. QQZ and QNW are the guarantors of this work; as such, they had full access to all the data in the study and take responsibility for the integrity of the data and the accuracy of the data analysis.

## Trial registration

This study was registered in the Chinese Clinical Trial Registry (ChiCTR2500105645) on July 8, 2025.
